# Distinct Trajectories of Recovery Within the First Year After Hip Fracture

**DOI:** 10.1093/geroni/igaa057.711

**Published:** 2020-12-16

**Authors:** Heather Mutchie, Denise Orwig, Ann Gruber-Baldini

**Affiliations:** 1 University of Maryland Baltimore, Baltimore, Maryland, United States; 2 Division of Gerontology, Baltimore, Maryland, United States; 3 University of Maryland School of Medicine, Baltimore, Maryland, United States

## Abstract

Hip fracture patients experience heterogenous pathways of recovery. Knowing about these different groups can guide rehabilitation efforts. The sample was 339 hip fracture patients age 65+ recruited to the Baltimore Hip Studies 7th cohort [2006-2011; men(n=168) women(n=171)] assessed at baseline, 2.6, and 12 months. Group based trajectory modeling assessed participants’ cognitive and functional recovery trajectories as measured by the Modified Mini-Mental State Examination(3MS;0-100), Hooper Visual Organization Task(HVOT,0-30), Trail-Making Task(TrailsA,B:0-301s), Short Physical Performance Battery(SPPB,0-12), Lower Extremity Physical Activities of Daily Living(LPADL,0-12), and physical activity (YALE, hours/week). Participants were on average 80.91(7.88) years old with 13(3.41) years of education. Two groups were identified for Trails A [Consistent high performance (85.3%), declined performance (14.6%)] and YALE [low exercise(94.9%), small decline(5.0%)]. Three groups were identified for 3MS [consistently high(76.3%), consistently borderline(17.1%), consistently low-performing(6.4%)] and SPPB [failure(19.3%), low-performing with small improvement(55.7%), moderate improvement(24.9%)]. Four groups were identified for HVOT[low scoring(14.2%), mid-range(31.9%), borderline(32.4%), well-performing(21.3%)], Trails B [consistent high-performance(40.2%), improved(18.6%), declined(12.0%), consistent failure(29.1%)], and LPADL[low-performing(27.0%), small improvement(50.0%), dramatic improvement(17.0%), high-performing(5.7%)]. The polynomial functions of the trajectory groups were primarily quadratic with some cubic functions best representing the recovery trajectories. Many of the groups have similar trajectories but at different magnitudes. For most tests performance at baseline was able to differentiate performance at 1 year. These data show that there are very few changes in high or low performing groups, but mid-range participants can experience volatility.

